# SAPHO Syndrome Complicated by Ankylosing Spondylitis Successfully Treated With Tofacitinib: A Case Report

**DOI:** 10.3389/fimmu.2022.911922

**Published:** 2022-05-25

**Authors:** Fangfang Yuan, Jing Luo, Qiong Yang

**Affiliations:** ^1^ Department of Rheumatism and Immunology, Ningbo No. 6 Hospital, Ningbo, China; ^2^ Department of Pharmacy, Ningbo No. 6 Hospital, Ningbo, China

**Keywords:** SAPHO syndrome, ankylosing spondylitis, tofacitinib, adalimumab, case report

## Abstract

Synovitis, acne, pustulosis, hyperostosis, and osteitis (SAPHO) syndrome, a type of chronic inflammatory disease, is rare and difficult to treat. Osteoarthropathy with skin involvement is the primary clinical manifestation of SAPHO syndrome. The unknown pathogenesis of SAPHO syndrome is speculated to be related to individual genetic differences, immune levels, microorganisms, and environmental factors. Tofacitinib, a novel small-molecule Janus kinase (JAK) inhibitor, has been used to treat rheumatoid arthritis. However, it also has great potential for the treatment of other immune diseases, including SAPHO syndrome. A 36-year-old man with chest and back pain for more than two months was admitted to our hospital. After admission, the patient developed a pustular rash and enteritis. SAPHO syndrome was diagnosed based on the above clinical manifestations, computed tomography (CT), and bone scintigraphy findings. Notably, the patient also had ankylosing spondylitis. Tofacitinib significantly improved the patient’s skin symptoms while preventing worsening of chest and back pain when adalimumab was discontinued. We report the first case of ankylosing spondylitis with SAPHO syndrome. In addition, it is also the first successful treatment thereof with tofacitinib. We hope to provide valuable information regarding the pathogenesis and treatment of SAPHO syndrome in this case.

## Introduction

Due to its rarity, synovitis, acne, pustulosis, hyperostosis, and osteitis SAPHO syndrome has been misdiagnosed as other diseases, including aseptic osteomyelitis and sternoclavicular hypertrophy, until Chamot et al. described it as SAPHO syndrome in 1987 (1). However, the pathogenesis of SAPHO syndrome remains unclear. Immune dysfunction, microbial infection, and genetic factors may all contribute to SAPHO syndrome (2–4). Women have a higher incidence of SAPHO than men (5). Osteoarthropathy with skin involvement is the primary clinical manifestation of SAPHO syndrome. In addition to the above clinical features, imaging studies are necessary for the diagnosis of SAPHO syndrome, including computed tomography (CT) and bone scintigraphy. Currently, there is no standard treatment for SAPHO syndrome. The nonsteroidal anti-inflammatory drugs (NSAIDs) are often used as first-line drugs to control symptoms. The glucocorticoids and disease-modifying antirheumatic drugs (DMARDs) have been reported to be effective. And biologics have achieved good results in individual cases (6). In recent years, tofacitinib has shown great potential for the treatment of SAPHO syndrome (7). Tofacitinib is a novel small molecule biologic that acts Janus kinase (JAK). It modulates the JAK signaling pathway and affects cytokine signaling during immune responses. Ankylosing spondylitis (AS) is a chronic inflammatory disease that primarily affects the spine and sacroiliac joints. It mainly causes pain in the back, gluteal region, or lumbosacral area. Here, we describe an extremely rare case of SAPHO syndrome and AS and the first case of successful treatment thereof with tofacitinib.

## Case Description

A 36-year-old man who presented with a 9-year history of recurrent bilateral pain in the gluteal region accompanied by pain in the lumbosacral area was admitted to our hospital in July 2014. His medical history revealed that he was diagnosed with AS through a comprehensive clinical assessment in 2012. Celecoxib capsules (0.2 g po bi-diurnally) were taken for 2 years to control his symptoms. During the last 2 months of the 2-year treatment period, owing to irregular use, the condition worsened. Laboratory test results were as follows: human leukocyte antigen B27 (HLA-B27), positive; C-reactive protein (CRP), 23.7 mg/L; and erythrocyte sedimentation rate (ESR), 48 mm/h. Radiography showed grade 2 to 3 bilateral sacroiliitis and MRI of the spine showed inflammation at the attachment point (**Figure S1**). Based on the above results, ankylosing spondylitis diagnosis was established, and the presentation met the modified New York criteria.

During hospitalization, recombinant human tumor necrosis factor-α receptor II (IgG Fc fusion protein injection [YISAIPU^®^, 25 mg ih biweekly]) was administered to the patient. The patient’s pain gradually resolved, and the drug was discontinued after 1 year. In 2017, the patient suffered from recurrent pain in both hips and was treated again with recombinant human tumor necrosis factor-α receptor II (IgG Fc fusion protein for injection [YISAIPU, 25 mg ih biweekly) to control the condition, and the symptoms resolved. In 2019, the patient discontinued YISAIPU, and subsequently developed neck stiffness and pain, significantly limited mobility, and a scaling rash on the scalp, left calf, and feet, which did not improve after YISAIPU was re-administered. Therefore, adalimumab injection (40 mg ih once every 2 weeks) was administered. The patient’s neck pain and rash gradually subsided.

**Figure 1:**
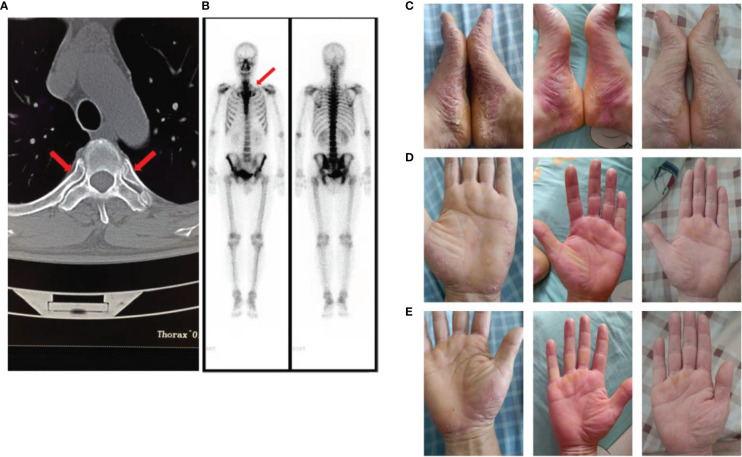
**(A)** The computed tomography image showed inflammatory changes of the breastbone; **(B)** The “bull’s head sign” on bony scintigraphy; **(C)** Palmoplantar pustulosis (PPP) on the patient’s palms and feet before using tofacitinib; **(D, E)**. PPP on the patient’s palms and feet after using tofacitinib for one month and two months.

In January 2022, the patient was re-admitted to our hospital for chest and back pain and difficulty in turning over. However, adalimumab did not relieve his symptoms. In addition, the patient developed a pustular rash on the scalp, left calf, and both feet, which was itchy and gradually increased in size. Other symptoms included obvious abdominal pain after eating and diarrhea. Laboratory test results were as follows: CRP, 66.2 mg/L and ESR, m/h. Electronic colonoscopy revealed ulcers in the terminal ileum and multiple ulcers in the colon and rectum. Free inflammatory exudates were also observed (**Figure S2**). The skin disease was diagnosed as palmoplantar pustulosis (PPP) and hidradenitis suppurativa through consultation with dermatologists. On imaging, CT showed inflammatory changes of the breastbone. Notably, the characteristic “bull’s head sign,” indicated by significant tracer enrichment in the front wall of the chest, was observed on bony scintigraphy. Through comprehensive clinical assessment, a diagnosis of SAPHO syndrome was established (**Figures 1A–C**).

**Figure 2:**
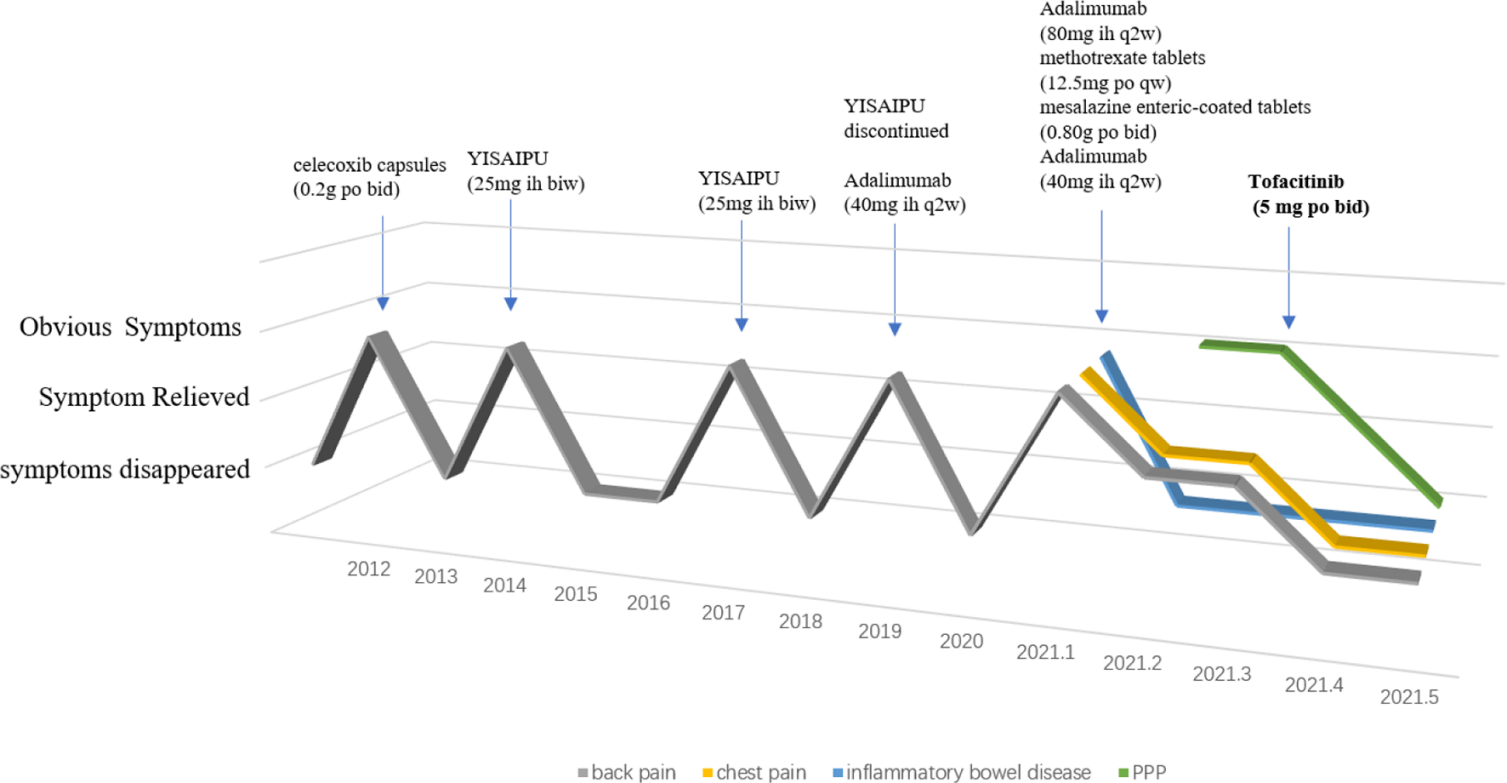
Treatment options and outcomes for patients from 2012 to 2021.

The patient was treated as follows: methotrexate tablets (12.5 mg po once a week) and mesalazine enteric-coated tablets (0.80 g po bi-diurnally) for 3 months, adalimumab injections (80 mg ih, once every 2 weeks) for 1 month, and adalimumab injection (40 mg ih, once every 2 weeks) for 3 months. The above treatment effectively relieved the patient’s chest and lower back pain, and diarrhea, but did not have any effect on the rash. After discussion with physicians, adalimumab (40 mg ih, once every 2 weeks) was discontinued and switched to tofacitinib (5 mg po bi-diurnally). The patient’s rash subsided significantly after 1 month (**Figures 1D, E**), and the chest and back pain was further relieved (**Figure 2**). The CRP level was 2.0 mg/L and ESR was 10 mm/h.

## Discussion

SAPHO syndrome has not been diagnosed using a single and accurate indicator. The diagnostic criteria proposed by Kahn et al. (8) in 1994 focuses on the skin and ossifying lesions. Pain and swelling of the anterior chest wall caused by osteitis or hyperostosis are often the main manifestations of SAHPO syndrome (9). Some patients also experience pain in the shoulder, sacroiliac, and small joints (phalanges) (10). PPP is concentrated on the palms and feet and is a cutaneous characteristic of SAPHO syndrome. In addition, imaging, including CT and bony scintigraphy, is extremely valuable for the diagnosis of SAPHO. The “bull’s head sign” on bony scintigraphy is considered a typical symptom of SAPHO syndrome but it does not appear in all patients (11). The pathogenesis of SAPHO syndrome is a complex process. Some immune cytokines, including serum interleukin 6 (IL-6), serum interleukin 8 (IL-8), and endothelin 1 (ET-1) (12), are currently reported to play a role in disease progression. SAPHO syndrome often induces other immune diseases, including psoriasis, Sjögren’s syndrome, and enteritis. In addition, the incidence of diabetes and depression is increased in patients with SAPHO syndrome (12). There is no standard treatment plan for SAPHO syndrome. Traditional antirheumatic treatments, particularly using methotrexate and sulfasalazine, have been reported to be effective. There have also been cases of successful treatment of SAPHO syndrome using biologics, such as infliximab, adalimumab, and tofacitinib.

In the present case, the patient had nearly all the typical clinical features of SAPHO syndrome: pain in the anterior chest wall, bone inflammation on CT, “bull’s head sign” on bony scintigraphy, inflammatory bowel disease, PPP on the skin, and hidradenitis suppurativa. After screening for infectious inflammation and bone tumors, the patient was diagnosed with SAPHO syndrome. However, the unique feature of this case is that the patient had AS. AS is a chronic disease in which the spine is the main lesion site, with the involvement of the sacroiliac joints. The comorbidities of SAPHO syndrome often occur after SAPHO syndrome, and only one case of coexistence of rheumatoid arthritis and SAPHO syndrome has been reported (13). SAPHO syndrome with AS has not been reported. The patient’s recurrent AS symptoms were relieved by adalimumab 2 years ago. With the development of SAPHO syndrome, the patient’s back pain reappeared. We speculated that SAPHO syndrome induced AS relapse. Therefore, adalimumab, which failed to prevent disease progression in the early stages of SAHPO syndrome, relieved the patient’s pain, but not the rash, during subsequent treatments. Pain and bowel disease may be the common effects of SAPHO and AS.

Compared to the pathogenesis of SAPHO syndrome, the pathogenesis of AS is clearer. However, research has not shown any shared genetic variants between SAPHO syndrome and AS (14). Among AS-related genes, HLA-27 is the most important genetic risk factor, and more than 80% of patients tested positive to HLA-B27 (15). However, among patients with SAPHO syndrome, the proportion of patients which showed HLA-B27 positivity decreased significantly (16). It is reasonable to infer that there are different molecular mechanisms between SAPHO syndrome and AS. However, with a deepening understanding of AS, the role of non-HLA proteins has been continuously revealed. Variations in non-HLA proteins interact with HLA alleles, resulting in the co-occurrence of different diseases in AS. Among these proteins, IL-17 plays a very important role. This cytokine is abundant in serum, synovial fluid, and the joints of AS patients (17). Inhibitors of IL-17 have been shown to be effective in the treatment of AS. Secukinumab is the first inhibitor of IL-17 approved for AS treatment (18). Notably, IL-17 also promotes the progression of SAPHO syndrome. Increased numbers of TH17 cells were found in the peripheral blood of patients with SAPHO syndrome (19). Multiple proteins, including MAPK1, SYK, ITB3 (ITGB3), PP2AA, and 2AAB, were differentially expressed in exosomes from patients with SAPHO syndrome. The action network composed of the above five proteins participate in the regulation of TNF-α and IL-17 signaling pathways (20). In addition, secukinumab has been shown to have a palliative effect on SAPHO syndrome (21). It can be seen that although SAHPO syndrome and AS have their own specific molecular mechanisms, there remain some overlapping signaling pathways, which we speculate is the basis for the coexistence of SAPHO and AS.

Owing to the patient’s refractory PPP, adalimumab was replaced with tofacitinib. Tofacitinib is a novel small-molecule inhibitor of the JAK signaling pathway, especially JAK1 and JAK3. It is used for the treatment of active rheumatoid arthritis in which methotrexate is insufficiently effective or intolerable. In recent years, tofacitinib has been found to be effective in treating psoriasis, AS, and Sjögren’s syndrome (22). Because of its downregulation of immune cytokines, including IL-6 and IL-8 (23), tofacitinib is also a potential treatment option for SAPHO syndrome. In addition, tofacitinib can affect the nuclear factor κB ligand (RANKL) pathway, thereby regulating osteoclast-mediated bone resorption (24). Four cases of successful SAPHO treatment using tofacitinib have been reported since tofacitinib was launched in China in 2017 (25–28). In 2020, a retrospective analysis of the efficacy of tofacitinib in 12 patients with SAPHO showed significant multidimensional improvements following treatment with tofacitinib (29). Concerning AS treatment, an intention-to-randomized controlled study also proved that tofacitinib was more effective than the placebo (22). In this case, the patient’s PPP improved rapidly after receiving tofacitinib. Notably, we observed that after the discontinuation of adalimumab, the patient’s back pain did not recur but it improved. It is possible that tofacitinib also relieved AS during SAPHO syndrome treatment. This suggests that there may be some overlap in the pathogenesis of AS and SAPHO syndromes. We described in detail a case and the treatment of SAPHO syndrome complicated by AS. We believe this report provides valuable information on the pathogenesis of SAPHO syndrome and the treatment options.

## Data Availability Statement

The original contributions presented in the study are included in the article/s **Supplementary Material**. Further inquiries can be directed to the corresponding author.

## Ethics Statement

Written informed consent was obtained from the individual(s) for the publication of any potentially identifiable images or data included in this article.

## Author Contributions

FY performed data analysis and wrote the paper. JL and QY provided clinical information and supervised the study. All authors contributed to the article and approved the submitted version.

## Conflict of Interest

The authors declare that the research was conducted in the absence of any commercial or financial relationships that could be construed as a potential conflict of interest.

## Publisher’s Note

All claims expressed in this article are solely those of the authors and do not necessarily represent those of their affiliated organizations, or those of the publisher, the editors and the reviewers. Any product that may be evaluated in this article, or claim that may be made by its manufacturer, is not guaranteed or endorsed by the publisher.
