# Supplementary material for: SAPHO Syndrome Complicated by Ankylosing Spondylitis Successfully Treated With Tofacitinib: A Case Report
Source: Front Immunol. 2022 May 25;13:911922. doi: 10.3389/fimmu.2022.911922 (PMC9174507; doi:10.3389/fimmu.2022.911922)
Supplement: Supplementary file 1 [file DataSheet_1.docx]

Supplementary material


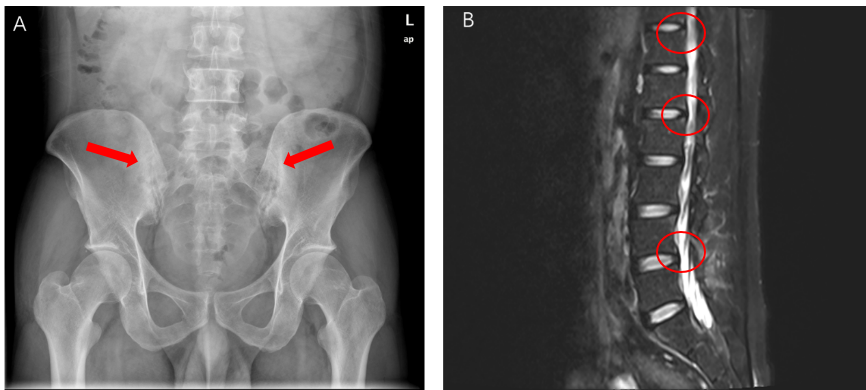


Figure S1: A. Radiography of the sacroiliac joints; B. MRI of the spine


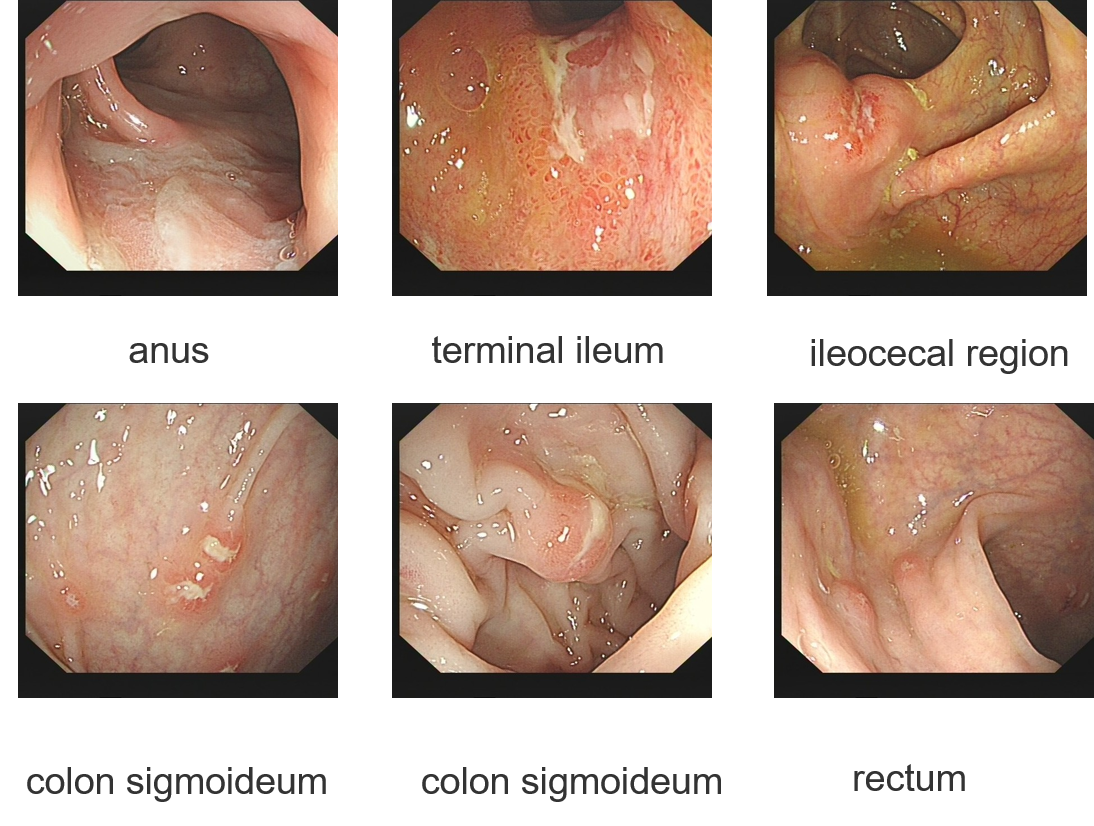


Figure S2: Colonoscopy images
